# Effects of AKT inhibitor therapy in response and resistance to BRAF inhibition in melanoma

**DOI:** 10.1186/1476-4598-13-83

**Published:** 2014-04-16

**Authors:** Amanda Lassen, Mohammad Atefi, Lidia Robert, Deborah JL Wong, Michael Cerniglia, Begonya Comin-Anduix, Antoni Ribas

**Affiliations:** 1 Department of Medicine, Division of Hematology-Oncology, David Geffen School of Medicine, University of California Los Angeles (UCLA), Los Angeles, CA, USA; 2 Department of Surgery, Division of Surgical Oncology, David Geffen School of Medicine, University of California Los Angeles (UCLA), Los Angeles, CA, USA; 3 Department of Molecular and Medical Pharmacology, David Geffen School of Medicine, University of California Los Angeles (UCLA), Los Angeles, CA, USA; 4 Jonsson Comprehensive Cancer Center (JCCC), UCLA, Los Angeles, CA, USA; 5 Department of Medicine, Division of Hematology-Oncology, 11-934 Factor Building, Jonsson Comprehensive Cancer Center at UCLA, 10833 Le Conte Avenue, Los Angeles, CA 90095-1782, USA

**Keywords:** Melanoma, AKT inhibitor, Dabrafenib, Combination therapy, Drug resistance

## Abstract

**Background:**

The clinical use of BRAF inhibitors for treatment of metastatic melanoma is limited by the development of drug resistance. In this study we investigated whether co-targeting the MAPK and the PI3K-AKT pathway can prevent emergence of resistance or provide additional growth inhibitory effects *in vitro*.

**Methods:**

Anti-tumor effects of the combination of the BRAF inhibitor (BRAFi) dabrafenib and GSK2141795B (AKTi) in a panel of 23 BRAF mutated melanoma cell lines were evaluated on growth inhibition by an ATP-based luminescent assay, on cell cycle and apoptosis by flow cytometry and on cell signaling by western blot. Moreover, we investigated the possibilities of delaying or reversing resistance or achieving further growth inhibition by combining AKTi with dabrafenib and/or the MEK inhibitor (MEKi) trametinib by using long term cultures.

**Results:**

More than 40% of the cell lines, including PTEN-/- and AKT mutants showed sensitivity to AKTi (IC_50_ < 1.5 μM). The combination of dabrafenib and AKTi synergistically potentiated growth inhibition in the majority of cell lines with IC_50_ > 5 nM dabrafenib. Combinatorial treatment induced apoptosis only in cell lines sensitive to AKTi. In long term cultures of a PTEN-/- cell line, combinatorial treatment with the MAPK inhibitors, dabrafenib and trametinib, and AKTi markedly delayed the emergence of drug resistance. Moreover, combining AKTi with the MAPK inhibitors from the beginning provided superior growth inhibitory effects compared to addition of AKTi upon development of resistance to MAPK inhibitors in this particular cell line.

**Conclusions:**

AKTi combined with BRAFi-based therapy may benefit patients with tumors harboring BRAF mutations and particularly PTEN deletions or AKT mutations.

## Introduction

BRAF inhibitors such as vemurafenib or dabrafenib efficiently block signaling downstream of the mutated BRAF^V600^ protein, which initially results in profound growth inhibition of the melanoma cells [1,2] and high frequency of tumor regression in the clinic [3,4]. However, the clinical use of these agents is limited by development of acquired drug resistance [5]. Accumulating data suggest that a single resistance mechanism does not account for acquired resistance to BRAF inhibitors – instead a diverse array of mutations and signaling alterations has been described. The best characterized core pathway resistance mechanism is reactivation of the MAPK pathway. This can be achieved by activating mutations in *NRAS*[6], amplification of the *BRAF*^*V600*^ gene or truncations in the BRAF^V600^ protein through alternative splicing resulting in lack of inhibition by the drug due to increased dimerization [7,8]. Activating mutations in *MEK* and overexpression of the Ser/Thr MAP kinase kinase kinases (MAP3K8, COT/Tpl2) has also been described in the context of BRAF inhibitor resistance [9-11]. A common feature for these MAPK reactivating resistance mechanisms is that they bypass inhibition of BRAF and thereby restore activation of ERK. Thus, blocking downstream MAPK pathway at the level of MEK, alone or in combination with BRAF inhibition could be a strategy to overcome this type of resistance and clinical trials addressing this issue are already ongoing [12]. It is highly likely that acquired resistance to the increasing use of dual BRAF and MEK inhibition for the upfront treatment of patients with metastatic melanoma may lead to increased reliance on MAPK-independent pathways during drug escape [13,14]. In this setting, oncogenic signaling can possibly be restored by enhanced signaling through the PI3K-AKT pathway. Over-activity of the PI3K-AKT pathway can be achieved by activating mutations in the signaling molecules, deletion of the phosphatase and tensin homolog (PTEN) or overexpression or over-activation of receptor tyrosine kinases (RTKs) such as the platelet derived growth factor beta (PDGFRβ) [6,15], the insulin-like growth factor receptor-1 (IGFR-1) [16] or the epidermal growth factor receptor (EGFR) [17] .

Given that the MAPK and the PI3K-AKT pathways are the predominant signaling pathways in melanoma and that MAPK-independent resistance to BRAF inhibitors can be mediated through enhancement of signaling through the PI3K-AKT pathway, it would be reasonable to combine a BRAF inhibitor with an inhibitor of the PI3K-AKT pathway to achieve synergistic antitumor activity [18-22]. This is further supported by the fact that these two pathways are connected in a complex network with extensive cross-talk and feedback loops operating at different levels [13,23-28].

In this study, we tested the hypothesis that combining the BRAF inhibitor dabrafenib, which recently has been approved for clinical use by the US Food and Drug Administration, with a novel AKT inhibitor tool compound GSK2141795B (AKTi), which is an analogue of the clinically tested AKT inhibitor GSK2141795, would have superior anti-tumor effects in *BRAF*^*V600*^ mutant melanoma cell lines compared to single agent dabrafenib. Furthermore, we investigated whether addition of the AKTi upon resistance to MAPK inhibitors could provide secondary responses, and whether upfront combination of dabrafenib, trametinib and AKTi could delay the emergence of drug resistance. Here we provide evidence that the combination of dabrafenib and AKTi synergistically inhibits proliferation in the majority of cell lines tested. Furthermore, we show that AKTi can delay the emergence of resistance to MAPK inhibitors and also provide further growth inhibition upon resistance to a combination of MAPK inhibitors in the only AKTi sensitive cell line tested in this study.

## Results

### Effects of single agent dabrafenib or AKTi on cell growth and cell signaling

**Figure 1:**
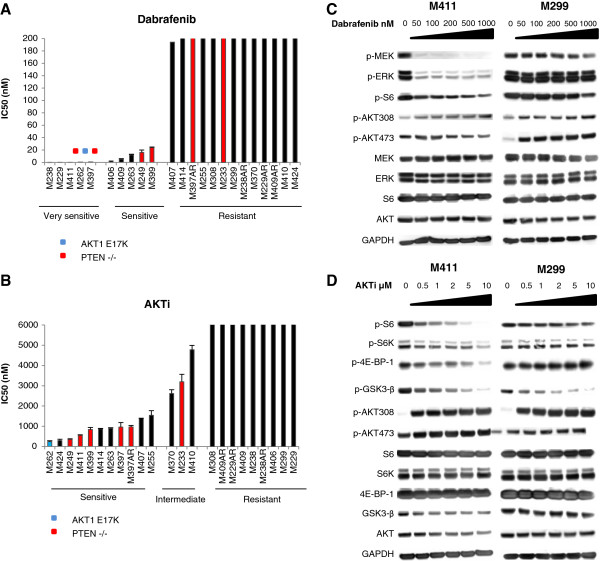
**IC50 values of *BRAF***^***V600 ***^**mutated melanoma cells after exposure to single agent dabrafenib or AKTi and the effects of the drugs on MAPK and the PI3K-AKT pathway.** A panel of 23 *BRAF*^*V600*^ mutant human melanoma cell lines was treated with serial dilutions (1-10,000 nM) of dabrafenib **(A)** or AKTi **(B)** for 72-120 hours to assess cell viability. The bars represent the average IC50 value of two or more independent experiments in duplicates and the error bars represent the SEM. Western blot analysis of phosphorylated proteins in MAPK and the PI3K-AKT pathway after 24 hours exposure to increasing concentrations of dabrafenib **(C)** or AKTi **(D)**.

In this study, a panel of 23 previously described [1,6] melanoma cell lines harboring *BRAF*^*V600*^ mutations (Table 1) was used to assess the effects of targeting the MAPK pathway and the PI3K-AKT signaling pathway. The panel included 19 drug naïve cell lines and four sub-lines (M229AR, M238AR, M397AR and M409AR) with acquired resistance to the BRAF inhibitor vemurafenib developed by continuous *in vitro* exposure to this drug [13]. The MAPK pathway was inhibited by the BRAF inhibitor dabrafenib and the PI3K-AKT pathway was inhibited by the AKT inhibitor GSK2141795B (AKTi). By performing growth assays (Additional file 1: Figure S1A) and arranging cell lines according to their IC50 values a cut-off of 100 nM for resistance to dabrafenib as single drug was determined on the basis of the natural gap in the IC50 values (Figure 1A). This divided the cell lines into two groups: sensitive (IC50 < 100 nM, 43%, 10 out of 23) and resistant (IC50 > 100 nM, 57%, 13 out of 23) to dabrafenib. The sensitive group could further be divided into two groups: very sensitive (IC50 < 1 nM) and sensitive (1 nM < IC50 < 100 nM). In 8 out of the 13 resistant cell lines (from M308 and to the right in Figure 1A), the IC50 was not achieved in the tested concentration range (e.g. IC50 < 10 μM). Based on the inhibitory effects of single agent AKTi and according to the calculated IC50 values for this inhibitor, cells lines were divided into three groups (Figure 1B): sensitive (IC50 < 1.5 μM), intermediate resistant (1.5 μM < IC50 < 5 μM) and resistant (IC50 > 5 μM). PTEN is a known negative regulator of the PI3K-AKT pathway and lack of expression or mutations in the protein can cause over activity of this pathway. Interestingly, most of the PTEN null cell lines were among the AKTi sensitive cell lines including M249, M411, M399, M397 and M397AR, indicated with red bars. However, M233 has homozygous *PTEN* loss but was less sensitive to AKTi. The only known *AKT* mutant in this series, M262 (*AKT1*^*E17K*^), was also found in the sensitive group (blue bar).

**Table 1:** Cell line characterization

	**BRAF**	**Other oncogenic events**
M229	BRAF V600E homozygous	BRAF amp., AKT amp., PTEN heterozygous, MITF amp.
M229AR9	BRAF V600E homozygous	BRAF amp., AKT amp., PTEN heterozygous, MITF amp.
M233	BRAF V600E heterozygous	BRAF amp., AKT1 amp., CCND1 amp., EGFR amp., CDKN2A homo, PTEN null
M238	BRAF V600E heterozygous	CDKN2A homozygous, PTEN heterozygous
M238AR	BRAF V600E heterozygous	CDKN2A homozygous, PTEN heterozygous
M249	BRAF V600E heterozygous	BRAF amp., MITF amp., AKT2 amp., PTEN null
M255	BRAF V600E heterozygous	AKT2 amp., CCND1 amp., EGFR amp., CDKN2A homozygous
M262	BRAF V600E homozygous	AKT1 E17K, AKT amp., EGFR amp., CDKN2A homozygous
M263	BRAF V600E heterozygous	CDKN2A heterozygous
M299	BRAF V600E heterozygous	
M308	BRAF V600E heterozygous	BRAF amp., MITF amp., AKT2 amp., EGFR amp., CDKN2A heterozygous
M370	BRAF V600E heterozygous	
M397	BRAF V600E heterozygous	PTEN null
M397AR	BRAF V600E heterozygous	PTEN null
M399	BRAF V600E heterozygous	PTEN null
M406	BRAF V600E heterozygous	N/T
M407	BRAF V600E heterozygous	N/T
M409	BRAF V600E heterozygous	N/T
M409AR	BRAF V600E heterozygous	N/T
M410	BRAF V600E heterozygous	N/T
M411	BRAF V600E homozygous	PTEN null
M414	BRAF V600K	
M424	BRAF V600K	
N/T: Not tested		

The efficacy of the drugs in inhibiting the signaling pathways was verified by western blot analysis of phosphorylated proteins (Figure 1C and 1D). Dabrafenib caused a clear reduction in p-MEK, p-ERK and p-S6 at a concentration as low as 50 nM in the dabrafenib sensitive cell line M411, whereas such reductions were not evident in the dabrafenib resistant cell line M299 (Figure 1C). AKTi caused a concentration dependent decrease in p-S6, p-4E-BP-1 and p-GSK-3β in the AKTi sensitive cell line M411. On the contrary, in the AKTi resistant cell line M299, AKTi only reduced p-GSK-3β (Figure 1D). In both cell lines, both drugs induced p-AKTs, suggesting activation of feedback mechanisms; however the induction of p-AKTs was more pronounced by AKTi.

### Combinatorial treatment with dabrafenib and AKTi enhances cell growth inhibition in dabrafenib sensitive and resistant cell lines

**Figure 2:**
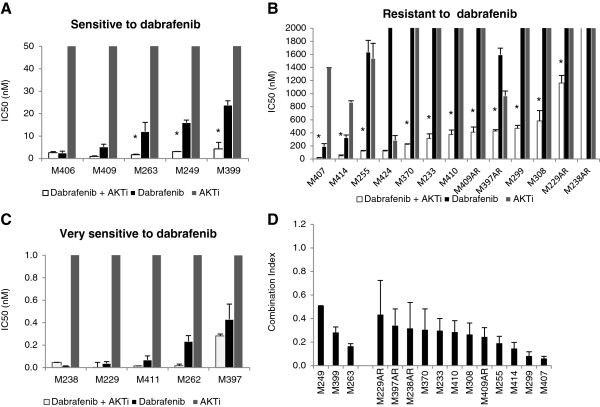
**Combination of dabrafenib with AKTi enhances cell growth inhibition in dabrafenib sensitive (A) and resistant cell lines (B) but not in very sensitive cell lines (C).** The cells were treated with increasing concentrations (1-10,000 nM) of single agent dabrafenib, single agent AKTi or the combination (1:1) for 72-120 hours to assess cell viability. Bars represent the average IC50 value of two or more independent experiments in duplicates and the error bars represent the SEM. **(D)** Combination index values (CI) for dabrafenib and AKTi. Values less than 1 indicates synergism, CI = 1 indicates an additive effect, and CI > 1 antagonism. The bars represent the average CI value of minimum two independent experiments and the error bars represent the SEM. * p < 0.05 when single agent dabrafenib was compared to combination treatment.

After evaluating the growth inhibition resulting from treatment with each drug alone, we explored whether blocking both pathways by the combination of dabrafenib and AKTi would enhance the growth inhibitory effects. The IC50 values for the combination of the two drugs (serial dilutions, in constant ratio to each other) were significantly lower compared to the IC50 values for each drug alone in the majority of cell lines sensitive and resistant to dabrafenib (Figure 2A and 2B). A similar trend was observed in the very sensitive group (Figure 2C). However, due to the extreme sensitivity of these cells to dabrafenib, additional growth inhibitory effect of AKTi was not as pronounced. In the sensitive group, the reduction in IC50 values ranged from 81% (M249) to 89% (M263) compared to the IC50 values for dabrafenib alone (Figure 2A). In the dabrafenib resistant group, the IC50 for dabrafenib was achieved in only four cell lines (e.g. IC50 > 10 μM). In these, the reduction in IC50 values with the combined treatment ranged from 73% (M397AR) to 93% (M255) (Figure 2B). In order to determine whether the enhanced growth inhibitory effects by the combined treatment were additive or synergistic, combination index values (CI) for the combination of the two drugs at IC50 were calculated by the Chou-Talalal method. The CI values showed synergistic effects (CI < 1) in all cell lines with a significant reduction in IC50 by the combined treatment (Figure 2D and Additional file 1: Figure S1B). However, at IC75 four (including M411 and M397) out of 5 cell lines in the very sensitive group showed synergism (data not shown).

### Basal levels of p-AKT in cell lines with differential sensitivity to dabrafenib and AKTi

**Figure 3:**
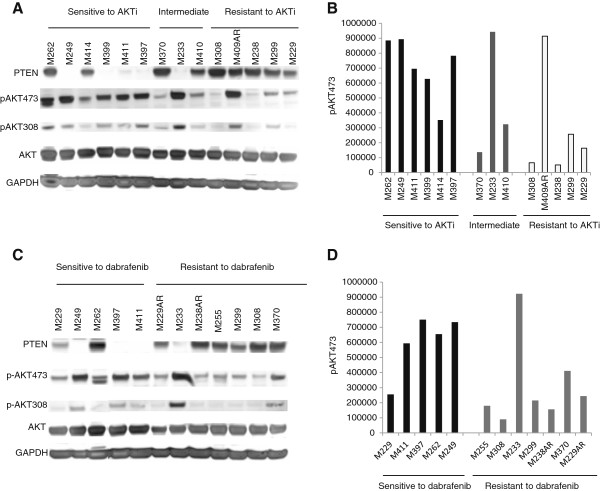
**Basal levels of p-AKT and PTEN status in cell lines with differential sensitivity to dabrafenib and AKTi.** Phosphorylation of AKT and PTEN status was determined by western blot analysis in a panel of cell lines with sensitivity or resistance to AKTi **(A + B)** or dabrafenib **(C + D)**. Quantitation of p-AKT was done by using image quant software. Total AKT and GAPDH served as loading controls.

Next we evaluated the responses seen in growth assays by quantitating basal levels of p-AKT in a representative panel of cell lines with differential sensitivity to single agent dabrafenib or AKTi (Figure 3). The first panel (Figure 3A + B) included 6 cell lines sensitive, 3 intermediate resistant and 5 cell lines resistant to AKTi. The data shows that p-AKTSer473 levels seem to be associated with responses to AKTi, where high level of p-AKT473 predicts sensitivity to AKTi, though with exception of M233 and M409AR. The second panel (Figure 3C + D) included 5 cell lines sensitive to dabrafenib and 7 cell lines resistant to this inhibitor. Surprisingly, in this panel the resistant cell lines did not express basal p-AKT473 at higher level compared with the sensitive cell lines, with the exception of M233. Moreover, in these panels, cell lines M249, M399, M411, M397 and M233 did not express PTEN.

### Changes in signaling through MAPK and PI3K-AKT pathways upon treatment with combination of dabrafenib and AKTi

**Figure 4:**
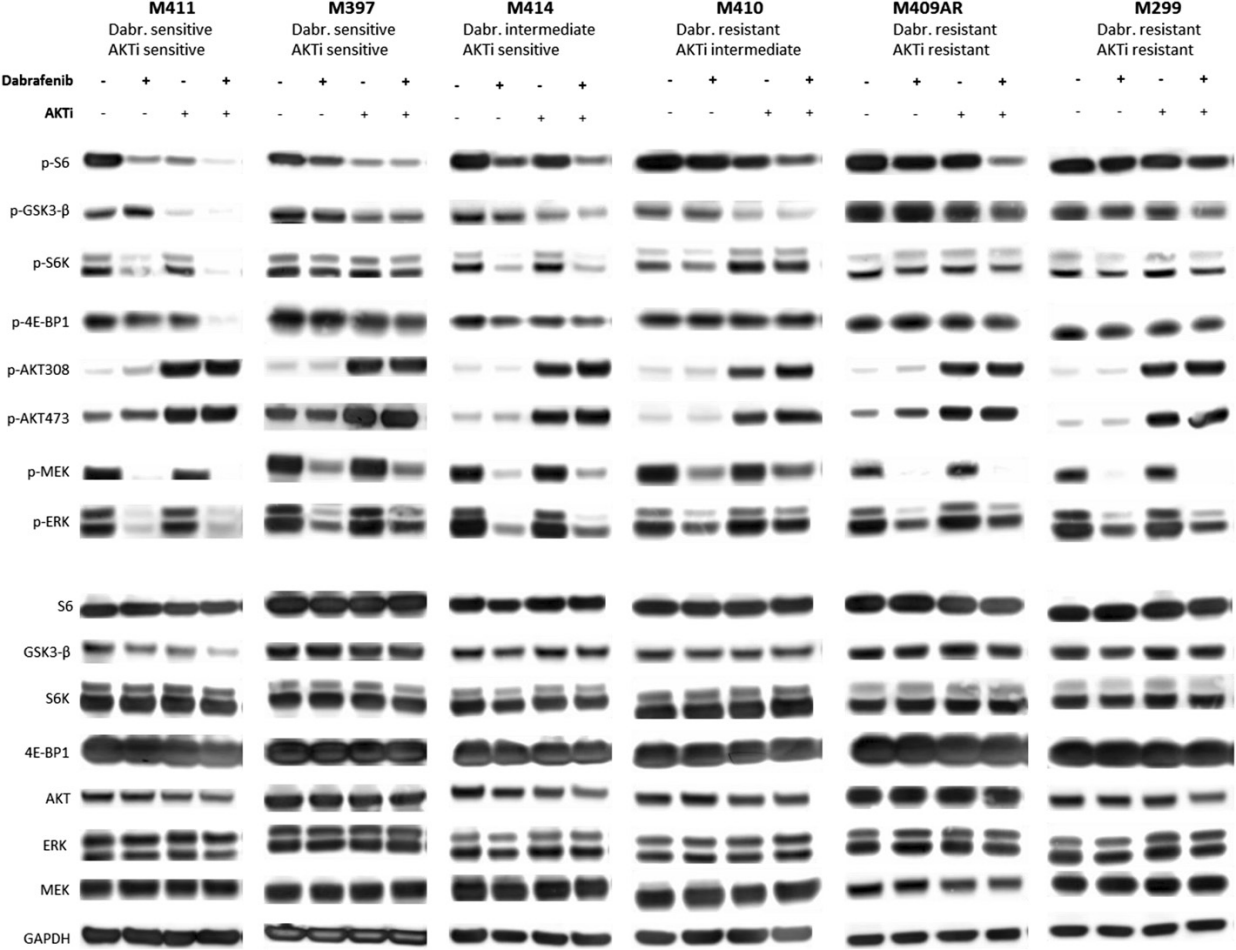
**Reduction of p-S6 with the combination of dabrafenib and AKTi in *BRAF***^***V600 ***^**mutated cell lines.** Western blot analysis of the effects of dabrafenib, AKTi or the combination on MAPK and the PI3K-AKT pathway in six representative *BRAF*^*V600*^ mutated cell lines with differential sensitivity to single agent AKTi and dabrafenib (Table 2). The analysis was performed after 24 hours exposure to DMSO (control), dabrafenib (50 nM), AKTi (2.5 μM) or the combination.

To further explore the effects of the two drugs, we selected 6 cell lines with differential sensitivity to single agent dabrafenib or AKTi (Table 2) and analyzed the impact on MAPK and PI3K-AKT signaling after 24 hours of exposure to the drugs either alone or in combination (Figure 4). In the dabrafenib and AKTi sensitive cell line M411 there was a clear reduction in p-S6, p-S6K, p-GSK-3β, p-MEK and p-ERK with one or the other drug as single agent. Combined treatment further reduced p-S6, p-GSK3-β, p-S6K and p-4E-BP-1 in comparison with each single agent treatment. In M397, single agent AKTi caused significant reduction in p-S6 and by addition of dabrafenib only a slight further decrease was achieved. The dabrafenib intermediate resistant, AKTi sensitive cell line M414 showed similar decreasing trends in p-S6 and p-GSK3-β, but less pronounced than in M411. In the dabrafenib resistant, AKTi intermediate sensitive cell line M410, AKTi alone caused some decrease in p-S6 and the combination resulted in further decrease. Noticeably, the presence of AKTi either alone or in combination increased the level of p-S6K in this cell line. In the two cell lines resistant to both drugs, M409AR and M299, a synergistic effect of combined treatment, assessed by reduction in p-S6, was observed only in M409AR. This finding is in agreement with the fact that growth inhibition with combined treatment of M409AR was superior to M299 (Additional file 1: Figure S1A). Despite resistance to dabrafenib, a decrease in p-MEK and p-ERK was seen in M410, M409AR and M299. Overall, reduction in p-S6 seemed to be the hallmark of the effects of single agent dabrafenib or AKTi or the combination (Additional file 2: Figure S2). In all the tested cell lines, AKTi alone or in combination induced the level of p-AKTs suggesting activation of a feedback mechanism.

**Table 2:** Drug sensitivities of 6 selected cell lines

	**Dabrafenib**	**AKTi**
	**Resistance: IC50 > 100nM**	**Resistance: IC50 > 1.5 μM**
M411	Sensitive	Sensitive
M397	Sensitive	Sensitive
M414	Intermediate	Sensitive
M410	Resistant	Intermediate
M409AR	Resistant	Resistant
M299	Resistant	Resistant

### Dabrafenib in combination with AKTi increases the subG1 population in AKTi sensitive cell lines and induces apoptosis

**Figure 5:**
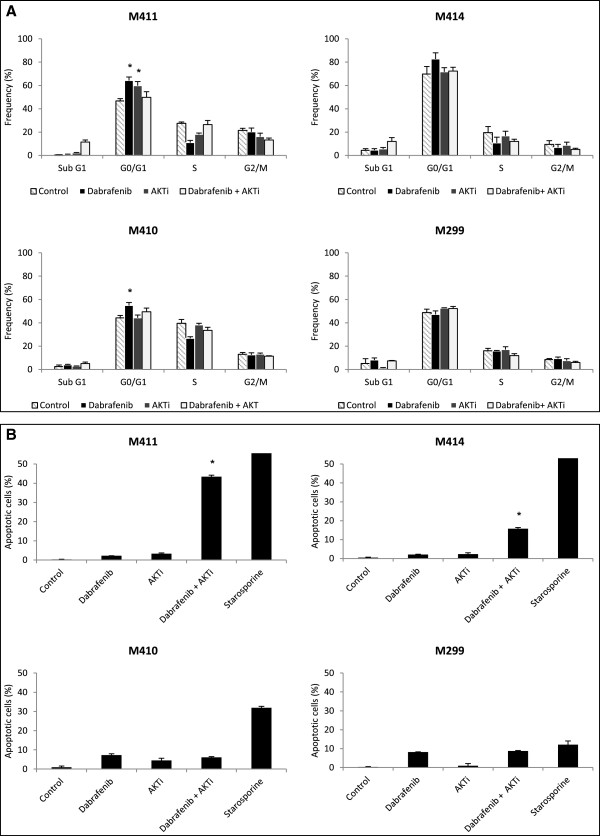
**Combined treatment with dabrafenib and AKTi increases the subG1 fraction and induces apoptosis in cell lines sensitive to AKTi as single agent.** Four representative melanoma cell lines with differential sensitivity to single agent AKTi and dabrafenib (Table 2) were cultured in DMSO (control), 1 μM staurosporine (positive control for apoptosis), 50 nM dabrafenib, 2.5 μM AKTi or the combination for 48 hours and stained with DAPI for cell cycle analysis **(A)** and anti-cleaved-PARP for apoptosis analysis **(B)**. Bars represent the average cell fractions of minimum two independent experiments and the error bars represent the SEM. * p < 0.05 when comparing single agent dabrafenib with the combination treatment.

To investigate whether dabrafenib or AKTi or the combination affect cell cycle, four representative cell lines (M411, M414, M410 and M299) with different dabrafenib and AKTi sensitivities (Table 2) were treated with DMSO (control) or either drug alone or in combination for 48 hours and stained with DAPI for cell cycle distribution analysis by flow cytometry. As expected [1,29], single agent dabrafenib compared with the control led to G0/G1 arrest, regardless of the sensitivity to this drug, except in the more resistant cell line M299 (Figure 5A). However, it should be noticed that the increase in G0/G1 fraction in M414 did not quite reach statistical significance (p = 0.055). AKTi as single drug led to significant G0/G1 arrest only in the relatively more AKTi sensitive cell line M411. The combined treatment did not change the fraction of cells in G0/G1 in any of the cell lines. More interesting, in the two AKTi sensitive cell lines, M411 and M414, the combined treatment resulted in a marked increase in the subG1 fraction suggesting that this treatment induced apoptosis. We further evaluated the apoptotic induction by detection of cleaved-PARP, which is a marker of cells undergoing late apoptosis [30,31] (Figure 5B). The cells were treated as mentioned above and treatment with staurosporine served as a positive control for apoptosis. Cells were stained with anti-cleaved-PARP antibody and analyzed by flow cytometry (Additional file 3: Figure S3). In agreement with the noticed increase in subG1 fraction by cell cycle analysis, combined treatment augmented apoptosis induction compared to single drug treatments only in the two AKTi sensitive cell lines M411 and M414. The induction was relatively more pronounced in cell line M411, which is sensitive to both drugs. These findings were confirmed using a cell death detection ELISA kit (Additional file 4: Figure S4).

### Addition of AKTi upon development of resistance to MAPK inhibitors can provide further growth inhibition in long term culture of a sensitive cell line, while triple treatment from the beginning delays the emergence of drug resistance

**Figure 6:**
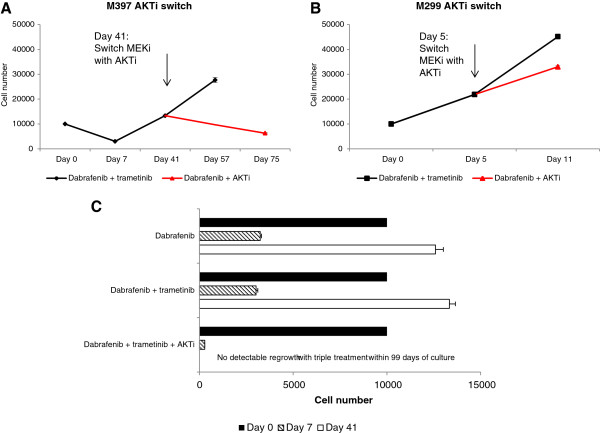
**Addition of AKTi upon development of resistance to MAPK inhibitors can provide further growth inhibition in long term culture, while triple treatment from the beginning delays the emergence of resistance.** The PTEN -/- cell line M397 **(A)** and M299 **(B)** were cultured in 96-well plates in the presence of 200 nM dabrafenib in combination with 2 nM trametinib. On day 41 or day 5 trametinib was replaced with AKTi (indicated by the arrow in the two cell lines respectively). **(C)** From day 0, M397 was cultured in the presence of 200 nM dabrafenib alone or in combination with 2 nM trametinib or in the presence of triple drugs; 200 nM dabrafenib, 2 nM trametinib and 2.5 uM AKTi. Cell numbers were determined by a MTS-based assay and use of a gradient with known number of cells which allowed the readout of each well to be calculated into a quantitative cell count (Additional file 5: Figure S5). The cell number for each time point represents the average cell number in 60 well replicates for the plates treated with dabrafenib and trametinib, and 120 replicate wells for the AKTi switch plates.

Whereas treatment with BRAF inhibitors at first results in tumor regression in most patients, it is a well-known fact that acquired drug resistance frequently develops [32]. To prevent or delay development of resistance to BRAFi, combinations of BRAFi and MEKi (MAPKi) are in clinical testing [12]. However, development of resistance to this MAPKi combination is predicted as well and addition of AKTi from the beginning or after the emergence of resistance as an alternative option has been suggested. By using long term *in vitro* culture as a model, we explored whether addition of AKTi upon emergence of resistance to dabrafenib in combination with the MEKi, trametinib, could provide further growth inhibition (Figure 6 A + B). The AKTi/MAPKi sensitive PTEN-/- cell line M397 and the AKTi/MAPKi resistant cell line M299 were cultured in 96-well plates in the presence of 200 nM dabrafenib in combination with 2 nM trametinib. Initially, growth of M397 was inhibited; after 7 days of culture a 70% reduction in cell number was achieved (Figure 6A). After a short period of 4-5 weeks the cells started to proliferate despite the presence of the drugs. On day 41, trametinib was replaced with 2.5 μM AKTi, which resulted in marked additional growth inhibition and decrease in cell numbers. As expected, from the beginning M299 continued growing despite the presence of the MAPK inhibitors. Therefore the experiment was performed in a shorter period of time with the switch from trametinib to AKTi on day 5, which only caused some reduction in growth rate (Figure 6B). Cell numbers were determined by an MTS-based assay and use of a gradient with known number of cells allowed the readout of each well to be calculated into a quantitative cell number (Additional file 5: Figure S5).

We then investigated whether a triple drug combination with AKTi, dabrafenib and trametinib from the beginning could delay the emergence of resistance using M397 in long term culture (Figure 6C). In this experiment, we treated the cells with either 200 nM dabrafenib as single drug or with 200 nM dabrafenib in combination with 2 nM trametinib or with 200 nM dabrafenib in combination with 2 nM trametinib and 2.5 μM AKTi. After 7 days of culture with dabrafenib alone or in combination with trametinib, the number of cells was reduced by 70%. However, despite the presence of the MAPK inhibitors, the cells started proliferating and within 41 days >12,000 cell/well on average were measured in the plates with single dabrafenib and in the plates with dabrafenib in combination with trametinib. Thus, addition of trametinib to dabrafenib did not delay the development of drug resistance, suggesting a non-MAPK pathway mechanism of resistance in this *PTEN* null cell line. In contrast, triple treatment reduced the cell number by >95% within 7 days. No signs of resistance or regrowth were evident within 99 days of culture with the triple inhibitor treatment which is in agreement with our assumption regarding the role of AKT pathway in resistance to MAPK inhibitors and possibility of using AKTi to avoid resistance.

## Discussion

Co-targeting the MAPK and the PI3K-AKT pathway is a compelling approach given the frequent cross-talk and regulating feedback loops between these two pathways [24,27]. Moreover, activation of the PI3K-AKT pathway has been suggested to mediate resistance to MAPK inhibitors [6,13,16,22,25,26], which strengthens the potential concept of inhibiting both pathways simultaneously. In our series, the single agent activity of the AKTi was more prominent in *PTEN* null cell lines and the only *AKT* mutant cell line, while the antitumor activity of dabrafenib was not negatively impacted by the presence of these alterations in the PI3K-AKT pathway. Our studies show that combining dabrafenib with AKTi had synergistic effects on growth inhibition in the majority of *BRAF*^*V600*^ mutant melanoma cell lines tested compared to single agent treatments, regardless of their sensitivity to the individual agents. The cell lines that did not show synergistic effects at IC50 belonged to the group very sensitive to single agent dabrafenib. The lack of synergism in this group is likely due to the fact that 50% growth inhibition was achieved at concentrations lower than 1 nM, which was the lowest concentration in the dilution series used. This makes the calculations of IC50 less reliable and an extension of the lower concentration range would likely result in measurable synergistic growth inhibitory effects. In fact, in 4 out of the 5 cell lines in question showed synergistic effects at IC75 (data not shown).

The finding that *PTEN* null and other cell lines expressing high levels of p-AKT are among the dabrafenib sensitive cell lines indicates that activation of the PI3K-AKT pathway is probably not a reason for the innate resistance to BRAF inhibition. Another explanation for this finding could be that, although these cell lines are primarily dependent on MAPK for their proliferation, they also to some extend are dependent on PI3K-AKT pathway for their proliferation and survival. This idea can be supported by the fact that in growth assays, these cell lines exhibit sensitivity to both dabrafenib and AKTi as single agents, and the combination treatment induced apoptosis in one tested PTEN null cell line (M411). Other studies exploring dual inhibition of the MAPK and the PI3K-AKT pathway using a different panel of inhibitors also found that combinations of MAPK and PI3-AKT pathway inhibitors augment induction of apoptosis in melanoma cells compared to single drug treatments [19,33,34]. Moreover, in cell lines with high levels of p-AKT, cell cycle analysis, apoptosis assay and long term drug treatment assays indicate the importance of both pathways and suggest that PI3K-AKT pathway gains higher importance in long term presence of BRAF inhibitors and during development of resistance to MAPK inhibitors.

In our studies, reduction in p-S6 seemed to be a good predictor of sensitivity to either of the single drugs or their combination. Reduction in p-S6 as a predictor of response to RAF and MEK inhibitors has also been described by others [35,36] and our data provides further evidence for the alleged cross-talk between the two pathways. Both sensitive and partially resistant cell lines to either drug exhibited decrease in p-S6 with single drugs or the combination, and a clear reduction was noticed in the double-resistant cell line M409AR with the combinatorial treatment. However, this was not observed in the cell line M299, which is even more resistant to both drugs and their combination. This suggests that reduction of p-S6 may be an indicator of response to either or dual targeting of MAPK and the PI3K-AKT pathway.

In the study of AKTi’s effects on the PI3K-AKT pathway, we observed a considerable increase in p-AKT at both phosphorylation sites namely T308 and S473. This induction suggests that the inhibition of AKT either abrogates a negative feedback loop or induces a positive regulation mechanism. Two different proteins have been reported to be responsible for phosphorylation at site T308 and S473, PDK1 acting from upstream and mTORC2 acting from downstream of AKT, respectively. A well-established feedback loop mediated by S6K inhibits the PI3K-AKT pathway through phosphorylation and inactivation of insulin receptor substrate-1 (IRS1), which activates PI3K [20,37]. Hence, inhibition of AKT would be expected to decrease phosphorylation of downstream S6K, consequently resulting in a feedback activation of PI3K with subsequently PDK1 activation and increased pAKT308 levels. However, in our study induction of pAKT308 was not consistently accompanied by a decrease in p-S6K. This could be explained by PDK1’s ability to phosphorylate S6K directly [38], and an induction in p-S6K by AKTi was in fact observed in M410.

Most patients with metastatic melanoma have early response with BRAF inhibitors as monotherapy, but acquired resistance frequently develops and the majority of patients experience relapse with a median of 6-7 months [4]. Supported by preclinical data showing that reactivation of the MAPK pathway often mediates acquired drug resistance [39,40], the effects of combination therapy with dabrafenib and the MEK inhibitor trametinib were evaluated in a phase I/II trial. It was found that BRAFi/MEKi combinatorial treatment improved the median progression-free survival and increased the response rate [12]. Though, as for monotherapy, resistance to the combined therapy invariably develops. Work from a recent publication by Wagle et. al suggest that most of the mechanisms of acquired resistance to combined BRAF and MEK inhibitor therapy represent alterations which retain the MAPK pathway active. Two of three reported MAPK alterations had previously been described in the context of resistance to RAF and MEK inhibitor monotherapy [41]. In addition to molecular changes in MAPK, genetic alterations up-regulating the PI3K-AKT pathway have been detected concurrently in the same tumor progressing on MAPK inhibitor therapy [39]. This suggests that BRAFi/MEKi combination therapy may lead to increased reliance on MAPK-independent pathways such as the PI3K-AKT pathway during drug escape. The results from our experiment modeling this scenario showed that replacement of trametinib with AKTi after the emergence of resistance to the combination of dabrafenib and trametinib had considerable growth inhibitory effects in the PTEN-/- AKTi sensitive cell line, M397. After removal of trametinib one can hypothesize that the melanoma cells would switch back to depend on BRAF independent MAPK pathway signaling, which naturally raises the thought of combining all three inhibitors; dabrafenib, trametinib and AKTi. Using the same cell line, our data demonstrates that triple combinatorial treatment can delay the emergence of drug resistance significantly. Cells cultured in the presence of dabrafenib and trametinib started re-growing within 4-5 weeks, while we were not able to detect any signs of resistance and regrowth in the presence of all three inhibitors within 99 days of culture.

## Conclusion

Overcoming resistance to BRAFi is a major problem in the treatment of metastatic melanoma. Multiple strategies including combinatorial therapies are evaluated in the attempt to solve this problem. Herein, we showed that combining the BRAF inhibitor dabrafenib with an AKTi potently inhibits growth in the majority of melanoma cell lines tested and induces cell death in a subset of cell lines. Moreover, AKT inhibition demonstrated ability to reverse acquired drug resistance to combination therapy with dabrafenib and trametinib in the single AKTi sensitive cell line that was tested (M397). Finally, triple drug administration delayed the emergence of drug resistance in that particular cell line. Thus, combining dabrafenib with an AKTi appears to be a promising strategy for more effective treatment of melanoma. This is the basis of a US cooperative group clinical trial (S1221, NCT 01902173), which has the goal of determining the safety of the combination of dabrafenib and the clinical grade AKTi GSK2141795, and early evidence of the antitumor activity of this combination in patients progressing on prior BRAFi-based therapy.

## Materials and methods

### Reagents

Dabrafenib (GSK2118436A), trametinib (GSK1120212B) and GSK2141795B (AKT inhibitor, AKTi) powder were obtained under a materials transfer agreement with GlaxoSmithKline (GSK, PA). The compounds were dissolved in dimethyl sulfioxide (DMSO, Sigma-Aldrich, St. Louis, MO) to a stock concentration of 10 mM.

### Cell lines and culturing

Human melanoma cell lines from the M series were established from patient’s biopsies under the University of California Los Angeles (UCLA) Institutional Review Board approval IRB#02-08-067. Cell lines with *in vitro* acquired resistance to vemurafenib were generated as previously described [13] and labeled as the parental cell line followed by “AR” for acquired resistance. Cells were cultured in RPMI 1640 with L-glutamine (Mediatech Inc., Manassas, VA) containing 10% fetal bovine serum (FBS, Omega Scientific, Tarzana, CA) and 1% penicillin, streptomycin and amphotericin (Omega Scientific). All cell lines were mycoplasma free when periodically tested using a Mycoalert Mycoplasma Detection Kit (Lonza, Rockland, ME). Cell cultures were maintained at 37°C in a humidified atmosphere of 5% CO_2_ and passaged into new flasks as needed. Information of the mutational status of the cell lines used in the study has been previously described [1].

### Growth assays

For short term growth assays, melanoma cell lines were seeded in 96-well plates (3000 cells/well). The following day, the cells were treated in duplicate with dabrafenib, AKTi or the combination in 10-fold serial dilutions starting from 10 μM for 72-120 hours depending on each cell line’s specific growth rate. Cell viability was measured by using the CellTiter-GLO® Luminescent Cell Viability assay (Promega, Madison, WI). The IC50 values were determined by interpolation from the dose-response curve. Each experiment was repeated at least three times and the average of minimum two is presented. In long term assays, cells were seeded in 96-well plates (10,000 cells/well). To study delays in emergence of resistance, the cells were treated with 200 nM dabrafenib (2 × IC80 for M397) alone or in combination with 2 nM trametinib (2 × IC80 for M397) or the combination of all three drugs including 2.5 μM AKTi. In another setup, cells were treated with dabrafenib and trametinib at the above mentioned concentrations and upon development of resistance to these two drugs, trametinib was replaced with 2.5 μM AKTi. Culture media containing the drugs was changed once a week. Growth of the cells was monitored and upon confluence of some wells, a gradient of the cells were plated to be used as a reference for the cell number. One hour before cell viability was determined using a tetrazolium compound [3-(4,5-dimethylthiazol-2-yl)-5-(3-carboxymethoxyphenyl)-2-(4-sulfophenyl)-2H-tetrazolium (MTS)-based colorimetric cell proliferation assay (Promega) the drug media was replaced with fresh culture media without drugs to eliminate measurement of absorbance of the drugs and to reduce the drugs impact on mitochondrial activity.

### Western blotting

Cells were seeded in 12-well plates at a density of 200,000 cells/well. The next day, the culture medium was replaced with media containing DMSO, 50 nM dabrafenib, 2.5 μM AKTi or the combination. After 24 hours, the media was removed and the cells were lysed directly in the wells for 15-20 min with lysis buffer (Pierce RIPA buffer, Thermo Scientific) containing phosphatase- and protease inhibitors (Sigma) and protein was extracted for western blot analysis as previously described [19]. Blots were blocked and probed with primary antibodies in 5% milk or 5% bovine serum albumin (BSA) in 1% Tween-20 phosphate-buffered saline (PBS-tween), washed with PBS-tween three times and incubated with horse-radish-linked secondary antibodies. Primary antibodies included p-AKT Ser473 and Thr308, AKT, p-S6K Thr389, S6K, p-S6 Ser235/236, S6, p-4EBP-1, 4EBP-1, p-GSK-3β, GSK-3β and GAPDH (all from Cell Signaling Technology, Danvers, MA). The immunoreactivity was visualized by use of an ECL-2 kit (Pierce, Thermo Scientific) and scanning of the blots by a Typhoon scanner (Amersham Biosciences Co, Piscataway, NJ). Quantification of protein levels from western blot analysis was done using ImageQuant software (version 5.2 Molecular dynamics).

### Cell cycle and apoptosis analysis

Cells were seeded at a density of 200,000 cells/well in 6-well plates. The following day, the culture medium was replaced by medium containing DMSO, 1 μM staurosporine (positive control for apoptosis), 50 nM dabrafenib, 2.5 μM AKTi or the combination. After 48 hours of exposure to the drugs, both adherent and floating cells were harvested by trypsinization and fixed for 20 minutes with Cytofix/Cytoperm solution (BD Biosciences, San Jose, CA). For apoptosis, cells were stained with Alexa Flour700 linked anti-cleaved-PARP antibody (5 μL/ 1 × 10^6^ cells, BD biosciences) for 30 minutes. Next, for cell cycle analysis, the cells were washed with Perm/Wash (BD Bioscience) before resuspended in 3 μM DAPI (Sigma-Aldrich) solution diluted in PBS containing 1% bovine serum albumin at a concentration of 1 × 10^6^ cells/mL. Flow cytometry was performed on a LSR-II (BD Bioscience) and data was analyzed using FlowJo (PC version 7, Tree Star Inc, Asland, OR).

### Cell death detection ELISA

Melanoma cell lines (5,000/well in 96-well plate) were treated in triplicate with DMSO, 50 nM dabrafenib, 2.5 μM AKTi or the combination for 48 hours. Apoptosis was quantified by anti-body-mediated capture and detection of cytoplasmic mononucleosome- and oligonucleosome-associated histone-DNA complexes (Cell death Detection ELISA plus kit, Roche) according to the manufacturer’s instructions. Results were expressed as the average absorbance value of triplicates.

### Statistical analysis

IC50 values were calculated on the basis of the growth inhibition curves and define the concentration of drugs that resulted in 50% growth inhibition. Synergistic, additive or antagonistic effects of the drug combinations were determined by the use of the combination index (CI) method of Chou and Talalay using the Calcusyn software (version 2.0 Biosoft, Cambrigde,UK). Any CI values less than 1 indicates synergism, CI = 1 additive effect and CI > 1 antagonism. Error bars represent the standard error of the mean (SEM). A two-tailed unpaired t-test was used when applicable. P-values < 0.05 were considered to be statistically significant.

## Competing interests

AR has participated in scientific advisory boards from GSK. The honoraria from these boards are paid to Institutional accounts at UCLA.

## Authors’ contributions

AL, MA, DW, LR performed the experiments and AL co-wrote the manuscript. MA participated in the design of the study and in the interpretation of data and revised the manuscript critically. BCA designed the experiments performed by flow cytometry and MC participated in collecting the data. AR conceived the study and participated in its design and coordination and co-wrote the manuscript. All authors read and approved the final manuscript.

## Supplementary Material

Additional file 1: Figure S1 Effects of single agent dabrafenib, AKTi or the combination on cell proliferation and viability. Growth inhibition curves of melanoma cell lines with differential sensitivity to single agent dabrafenib and AKTi **(A)**. Cells were treated with increasing concentrations (1-10,000 nM) of dabrafenib, AKTi or the combination for 72-120 hours to assess cell viability. The graphs represent the average growth inhibition in percent of minimum two independent experiments in duplicates and the error bars represent SEM. Dose-effect parameters for the cell lines with synergy data **(B)**. The r value is the linear correlation coefficient of the median-effect plot while m denotes the shape of the dose-effect curves. Click here for file

Additional file 2: Figure S2 Quantitative analysis of p-S6 from western blots. Data corresponds to the p-S6 bands in the western blots presented in figure 4. The relative reduction in p-S6 in cell lines M411, M397, M414, M410 and M409AR corresponds with the response to the combined treatment, with the more sensitive cell line M411 showing the highest reduction and the resistant cell line M299 showing the slightest reduction in p-S6. Click here for file

Additional file 3: Figure S3 Anti-cleaved-PARP gating strategy. The fraction of anti-cleaved-PARP positive cells was determined by using an unstained control. Apoptosis induced by staurosporine was used as a positive control. The analysis was done by using FlowJo software (PC ver. 7). Click here for file

Additional file 4: Figure S4 Determining apoptosis using a cell death detection ELISA. Cells were treated with DMSO, 50 nM dabrafenib, 2.5 μM AKTi or the combination for 48 hours. The extent of apoptosis is reflected by the enrichment of nucleosomes in the cytoplasm, which was quantitated as the relative increase in absorbance (y-axis). The panel included one cell line sensitive to both dabrafenib and AKTi (M411), one cell line sensitive to AKTi but intermediate resistant to dabrafenib (M414) and one cell line demonstrating resistance to both drugs (M299). The bars represent the average absorbance of triplicates. Click here for file

Additional file 5: Figure S5 Example of a standard curve used for the calculations of cell number in long term culture. Cells were plated in 1:2 serial dilutions starting from 50,000 cells. The measured absorbance (using a MTS-based assay) from these wells were plotted against the known cell numbers and by use of the equation for the trend line through (0.0) the unknown cell numbers could be determined. Click here for file

## References

[B1] Sondergaard JN Nazarian R Wang Q Guo D Hsueh T Mok S Sazegar H MacConaill LE Barretina JG Kehoe SM Attar N von Euw E Zuckerman JE Chmielowski B Comin-Anduix B Koya RC Mischel PS Lo RS Ribas A Differential sensitivity of melanoma cell lines with BRAFV600E mutation to the specific Raf inhibitor PLX4032 J Transl Med 2010 8 39 10.1186/1479-5876-8-39 20406486 PMC2876068

[B2] Bollag G Hirth P Tsai J Zhang J Ibrahim PN Cho H Spevak W Zhang C Zhang Y Habets G Burton EA Wong B Tsang G West BL Powell B Shellooe R Marimuthu A Nguyen H Zhang KY Artis DR Schlessinger J Su F Higgins B Iyer R D'Andrea K Koehler A Stumm M Lin PS Lee RJ Grippo J Clinical efficacy of a RAF inhibitor needs broad target blockade in BRAF-mutant melanoma Nature 2010 467 596 599 10.1038/nature09454 20823850 PMC2948082

[B3] Hauschild A Grob J-J Demidov LV Jouary T Gutzmer R Millward M Rutkowski P Blank CU Miller WH Kaempgen E Martín-Algarra S Karaszewska B Mauch C Chiarion-Sileni V Martin A-M Swann S Haney P Mirakhur B Guckert ME Goodman V Chapman PB Dabrafenib in BRAF-mutated metastatic melanoma: a multicentre, open-label, phase 3 randomised controlled trial Lancet 2012 380 358 365 10.1016/S0140-6736(12)60868-X 22735384

[B4] Sosman JA Kim KB Schuchter L Gonzalez R Pavlick AC Weber JS McArthur GA Hutson TE Moschos SJ Flaherty KT Hersey P Kefford R Lawrence D Puzanov I Lewis KD Amaravadi RK Chmielowski B Lawrence HJ Shyr Y Ye F Li J Nolop KB Lee RJ Joe AK Ribas A Survival in BRAF V600-mutant advanced melanoma treated with vemurafenib N Engl J Med 2012 366 707 714 10.1056/NEJMoa1112302 22356324 PMC3724515

[B5] Fedorenko IV Paraiso KH Smalley KS Acquired and intrinsic BRAF inhibitor resistance in BRAF V600E mutant melanoma Biochem Pharmacol 2011 82 201 209 10.1016/j.bcp.2011.05.015 21635872 PMC4001781

[B6] Nazarian R Shi H Wang Q Kong X Koya RC Lee H Chen Z Lee MK Attar N Sazegar H Chodon T Nelson SF McArthur G Sosman JA Ribas A Lo RS Melanomas acquire resistance to B-RAF(V600E) inhibition by RTK or N-RAS upregulation Nature 2010 468 973 977 10.1038/nature09626 21107323 PMC3143360

[B7] Shi H Moriceau G Kong X Lee MK Lee H Koya RC Ng C Chodon T Scolyer RA Dahlman KB Sosman JA Kefford RF Long GV Nelson SF Ribas A Lo RS Melanoma whole-exome sequencing identifies (V600E)B-RAF amplification-mediated acquired B-RAF inhibitor resistance Nat Commun 2012 3 724 22395615 10.1038/ncomms1727 PMC3530385

[B8] Poulikakos PI Persaud Y Janakiraman M Kong X Ng C Moriceau G Shi H Atefi M Titz B Gabay MT Salton M Dahlman KB Tadi M Wargo JA Flaherty KT Kelley MC Misteli T Chapman PB Sosman JA Graeber TG Ribas A Lo RS Rosen N Solit DB RAF inhibitor resistance is mediated by dimerization of aberrantly spliced BRAF(V600E) Nature 2011 480 387 390 10.1038/nature10662 22113612 PMC3266695

[B9] Johannessen CM Boehm JS Kim SY Thomas SR Wardwell L Johnson LA Emery CM Stransky N Cogdill AP Barretina J Caponigro G Hieronymus H Murray RR Salehi-Ashtiani K Hill DE Vidal M Zhao JJ Yang X Alkan O Kim S Harris JL Wilson CJ Myer VE Finan PM Root DE Roberts TM Golub T Flaherty KT Dummer R Weber BL COT drives resistance to RAF inhibition through MAP kinase pathway reactivation Nature 2010 468 968 972 10.1038/nature09627 21107320 PMC3058384

[B10] Wagle N Emery C Berger MF Davis MJ Sawyer A Pochanard P Kehoe SM Johannessen CM Macconaill LE Hahn WC Meyerson M Garraway LA Dissecting therapeutic resistance to RAF inhibition in melanoma by tumor genomic profiling J Clin Oncol 2011 29 3085 3096 10.1200/JCO.2010.33.2312 21383288 PMC3157968

[B11] Emery CM Vijayendran KG Zipser MC Sawyer AM Niu L Kim JJ Hatton C Chopra R Oberholzer PA Karpova MB MacConaill LE Zhang J Gray NS Sellers WR Dummer R Garraway LA MEK1 mutations confer resistance to MEK and B-RAF inhibition Proc Natl Acad Sci U S A 2009 106 20411 20416 10.1073/pnas.0905833106 19915144 PMC2777185

[B12] Flaherty KT Infante JR Daud A Gonzalez R Kefford RF Sosman J Hamid O Schuchter L Cebon J Ibrahim N Kudchadkar R Burris HA 3rd Falchook G Algazi A Lewis K Long GV Puzanov I Lebowitz P Singh A Little S Sun P Allred A Ouellet D Kim KB Patel K Weber J Combined BRAF and MEK inhibition in melanoma with BRAF V600 mutations N Engl J Med 2012 367 1694 1703 10.1056/NEJMoa1210093 23020132 PMC3549295

[B13] Atefi M von Euw E Attar N Ng C Chu C Guo D Nazarian R Chmielowski B Glaspy JA Comin-Anduix B Mischel PS Lo RS Ribas A Reversing melanoma cross-resistance to BRAF and MEK inhibitors by co-targeting the AKT/mTOR pathway PLoS One 2011 6 e28973 10.1371/journal.pone.0028973 22194965 PMC3237573

[B14] Gowrishankar K Snoyman S Pupo GM Becker TM Kefford RF Rizos H Acquired resistance to BRAF inhibition can confer cross-resistance to combined BRAF/MEK inhibition J Invest Dermatol 2012 132 1850 1859 10.1038/jid.2012.63 22437314

[B15] Shi H Kong X Ribas A Lo RS Combinatorial treatments that overcome PDGFRbeta-driven resistance of melanoma cells to V600EB-RAF inhibition Cancer Res 2011 71 5067 5074 10.1158/0008-5472.CAN-11-0140 21803746 PMC3149831

[B16] Villanueva J Vultur A Lee JT Somasundaram R Fukunaga-Kalabis M Cipolla AK Wubbenhorst B Xu X Gimotty PA Kee D Santiago-Walker AE Letrero R D'Andrea K Pushparajan A Hayden JE Brown KD Laquerre S McArthur GA Sosman JA Nathanson KL Herlyn M Acquired resistance to BRAF inhibitors mediated by a RAF kinase switch in melanoma can be overcome by cotargeting MEK and IGF-1R/PI3K Cancer Cell 2010 18 683 695 10.1016/j.ccr.2010.11.023 21156289 PMC3026446

[B17] Girotti MR Pedersen M Sanchez-Laorden B Viros A Turajlic S Niculescu-Duvaz D Zambon A Sinclair J Hayes A Gore M Lorigan P Springer C Larkin J Jorgensen C Marais R Inhibiting EGF receptor or SRC family kinase signaling overcomes BRAF inhibitor resistance in melanoma Cancer Discov 2013 3 158 167 10.1158/2159-8290.CD-12-0386 23242808 PMC5321574

[B18] Meier F Schittek B Busch S Garbe C Smalley K Satyamoorthy K Li G Herlyn M The RAS/RAF/MEK/ERK and PI3K/AKT signaling pathways present molecular targets for the effective treatment of advanced melanoma Front Biosci 2005 10 2986 3001 10.2741/1755 15970553

[B19] Lasithiotakis KG Sinnberg TW Schittek B Flaherty KT Kulms D Maczey E Garbe C Meier FE Combined inhibition of MAPK and mTOR signaling inhibits growth, induces cell death, and abrogates invasive growth of melanoma cells J Invest Dermatol 2008 128 2013 2023 10.1038/jid.2008.44 18323781

[B20] Carracedo A Ma L Teruya-Feldstein J Rojo F Salmena L Alimonti A Egia A Sasaki AT Thomas G Kozma SC Papa A Nardella C Cantley LC Baselga J Pandolfi PP Inhibition of mTORC1 leads to MAPK pathway activation through a PI3K-dependent feedback loop in human cancer J Clin Invest 2008 118 3065 3074 18725988 10.1172/JCI34739 PMC2518073

[B21] Shimizu T Tolcher AW Papadopoulos KP Beeram M Rasco DW Smith LS Gunn S Smetzer L Mays TA Kaiser B Wick MJ Alvarez C Cavazos A Mangold GL Patnaik A The clinical effect of the dual-targeting strategy involving PI3K/AKT/mTOR and RAS/MEK/ERK pathways in patients with advanced cancer Clin Cancer Res 2012 18 2316 2325 10.1158/1078-0432.CCR-11-2381 22261800

[B22] Su F Bradley WD Wang Q Yang H Xu L Higgins B Kolinsky K Packman K Kim MJ Trunzer K Lee RJ Schostack K Carter J Albert T Germer S Rosinski J Martin M Simcox ME Lestini B Heimbrook D Bollag G Resistance to selective BRAF inhibition can be mediated by modest upstream pathway activation Cancer Res 2012 72 969 978 10.1158/0008-5472.CAN-11-1875 22205714

[B23] Ciuffreda L Di Sanza C Cesta Incani U Eramo A Desideri M Biagioni F Passeri D Falcone I Sette G Bergamo P Anichini A Sabapathy K McCubrey JA Ricciardi MR Tafuri A Blandino G Orlandi A De Maria R Cognetti F Del Bufalo D Milella M The mitogen-activated protein kinase (MAPK) cascade controls phosphatase and tensin homolog (PTEN) expression through multiple mechanisms J Mol Med (Berl) 2012 90 667 679 10.1007/s00109-011-0844-1 22215152

[B24] Aksamitiene E Kiyatkin A Kholodenko BN Cross-talk between mitogenic Ras/MAPK and survival PI3K/Akt pathways: a fine balance Biochem Soc Trans 2012 40 139 146 10.1042/BST20110609 22260680

[B25] Chen B Tardell C Higgins B Packman K Boylan JF Niu H BRAFV600E negatively regulates the AKT pathway in melanoma cell lines PLoS One 2012 7 e42598 10.1371/journal.pone.0042598 22880048 PMC3411810

[B26] Gopal YN Deng W Woodman SE Komurov K Ram P Smith PD Davies MA Basal and treatment-induced activation of AKT mediates resistance to cell death by AZD6244 (ARRY-142886) in Braf-mutant human cutaneous melanoma cells Cancer Res 2010 70 8736 8747 10.1158/0008-5472.CAN-10-0902 20959481 PMC4286702

[B27] Carracedo A Pandolfi PP The PTEN-PI3K pathway: of feedbacks and cross-talks Oncogene 2008 27 5527 5541 10.1038/onc.2008.247 18794886

[B28] McCubrey JA Steelman LS Abrams SL Lee JT Chang F Bertrand FE Navolanic PM Terrian DM Franklin RA D'Assoro AB Salisbury JL Mazzarino MC Stivala F Libra M Roles of the RAF/MEK/ERK and PI3K/PTEN/AKT pathways in malignant transformation and drug resistance Adv Enzyme Regul 2006 46 249 279 10.1016/j.advenzreg.2006.01.004 16854453

[B29] Niehr F von Euw E Attar N Guo D Matsunaga D Sazegar H Ng C Glaspy JA Recio JA Lo RS Mischel PS Comin-Anduix B Ribas A Combination therapy with vemurafenib (PLX4032/RG7204) and metformin in melanoma cell lines with distinct driver mutations J Transl Med 2011 9 76 10.1186/1479-5876-9-76 21609436 PMC3152784

[B30] Oliver FJ de la Rubia G Rolli V Ruiz-Ruiz MC de Murcia G Murcia JM Importance of poly(ADP-ribose) polymerase and its cleavage in apoptosis. Lesson from an uncleavable mutant J Biol Chem 1998 273 33533 33539 10.1074/jbc.273.50.33533 9837934

[B31] Lazebnik YA Kaufmann SH Desnoyers S Poirier GG Earnshaw WC Cleavage of poly(ADP-ribose) polymerase by a proteinase with properties like ICE Nature 1994 371 346 347 10.1038/371346a0 8090205

[B32] Flaherty KT Puzanov I Kim KB Ribas A McArthur GA Sosman JA O'Dwyer PJ Lee RJ Grippo JF Nolop K Chapman PB Inhibition of mutated, activated BRAF in metastatic melanoma N Engl J Med 2010 363 809 819 10.1056/NEJMoa1002011 20818844 PMC3724529

[B33] Meier F Busch S Lasithiotakis K Kulms D Garbe C Maczey E Herlyn M Schittek B Combined targeting of MAPK and AKT signalling pathways is a promising strategy for melanoma treatment Br J Dermatol 2007 156 1204 1213 10.1111/j.1365-2133.2007.07821.x 17388918

[B34] Smalley KS Haass NK Brafford PA Lioni M Flaherty KT Herlyn M Multiple signaling pathways must be targeted to overcome drug resistance in cell lines derived from melanoma metastases Mol Cancer Ther 2006 5 1136 1144 16731745 10.1158/1535-7163.MCT-06-0084

[B35] Corcoran RB Rothenberg SM Hata AN Faber AC Piris A Nazarian RM Brown RD Godfrey JT Winokur D Walsh J Mino-Kenudson M Maheswaran S Settleman J Wargo JA Flaherty KT Haber DA Engelman JA TORC1 Suppression Predicts Responsiveness to RAF and MEK Inhibition in BRAF-Mutant Melanoma Sci Transl Med 2013 5 196ra198 10.1126/scitranslmed.3005753 PMC3867020 23903755

[B36] Deng W Gopal YN Scott A Chen G Woodman SE Davies MA Role and therapeutic potential of PI3K-mTOR signaling in de novo resistance to BRAF inhibition Pigment Cell Melanoma Res 2012 25 248 258 10.1111/j.1755-148X.2011.00950.x 22171948

[B37] Harrington LS Findlay GM Gray A Tolkacheva T Wigfield S Rebholz H Barnett J Leslie NR Cheng S Shepherd PR Gout I Downes CP Lamb RF The TSC1-2 tumor suppressor controls insulin-PI3K signaling via regulation of IRS proteins J Cell Biol 2004 166 213 223 10.1083/jcb.200403069 15249583 PMC2172316

[B38] Mora A Komander D van Aalten DM Alessi DR PDK1, the master regulator of AGC kinase signal transduction Semin Cell Dev Biol 2004 15 161 170 10.1016/j.semcdb.2003.12.022 15209375

[B39] Shi H Hugo W Kong X Hong A Koya RC Moriceau G Chodon T Guo R Johnson DB Dahlman KB Kelley MC Kefford RF Chmielowski B Glaspy JA Sosman JA van Baren N Long GV Ribas A Lo RS Acquired Resistance and Clonal Evolution in Melanoma during BRAF Inhibitor Therapy Cancer Discov 2014 4 80 93 10.1158/2159-8290.CD-13-0642 24265155 PMC3936420

[B40] Van Allen EM Wagle N Sucker A Treacy DJ Johannessen CM Goetz EM Place CS Taylor-Weiner A Whittaker S Kryukov GV Hodis E Rosenberg M McKenna A Cibulskis K Farlow D Zimmer L Hillen U Gutzmer R Goldinger SM Ugurel S Gogas HJ Egberts F Berking C Trefzer U Loquai C Weide B Hassel JC Gabriel SB Carter SL Getz G The Genetic Landscape of Clinical Resistance to RAF Inhibition in Metastatic Melanoma Cancer Discov 2014 4 94 109 10.1158/2159-8290.CD-13-0617 24265153 PMC3947264

[B41] Wagle N Van Allen EM Treacy DJ Frederick DT Cooper ZA Taylor-Weiner A Rosenberg M Goetz EM Sullivan RJ Farlow DN Friedrich DC Anderka K Perrin D Johannessen CM McKenna A Cibulskis K Kryukov G Hodis E Lawrence DP Fisher S Getz G Gabriel SB Carter SL Flaherty KT Wargo JA Garraway LA MAP Kinase Pathway Alterations in BRAF-Mutant Melanoma Patients with Acquired Resistance to Combined RAF/MEK Inhibition Cancer Discov 2014 4 61 68 10.1158/2159-8290.CD-13-0631 24265154 PMC3947296

