# Supplementary material for: Effects of AKT inhibitor therapy in response and resistance to BRAF inhibition in melanoma
Source: Mol Cancer. 2014 Apr 16;13:83. doi: 10.1186/1476-4598-13-83 (PMC4021505; doi:10.1186/1476-4598-13-83)
Supplement: Additional file 1: Figure S1 — Effects of single agent dabrafenib, AKTi or the combination on cell proliferation and viability. Growth inhibition curves of melanoma cell lines with differential sensitivity to single agent dabrafenib and AKTi (A). Cells were treated with increasing concentrations (1-10,000 nM) of dabrafenib, AKTi or the combination for 72-120 hours to assess cell viability. The graphs represent the average growth inhibition in percent of minimum two independent experiments in duplicates and the error bars represent SEM. Dose-effect parameters for the cell lines with synergy data (B). The r value is the linear correlation coefficient of the median-effect plot while m denotes the shape of the dose-effect curves. [file 1476-4598-13-83-S1.pdf]

Figure S1
1/4

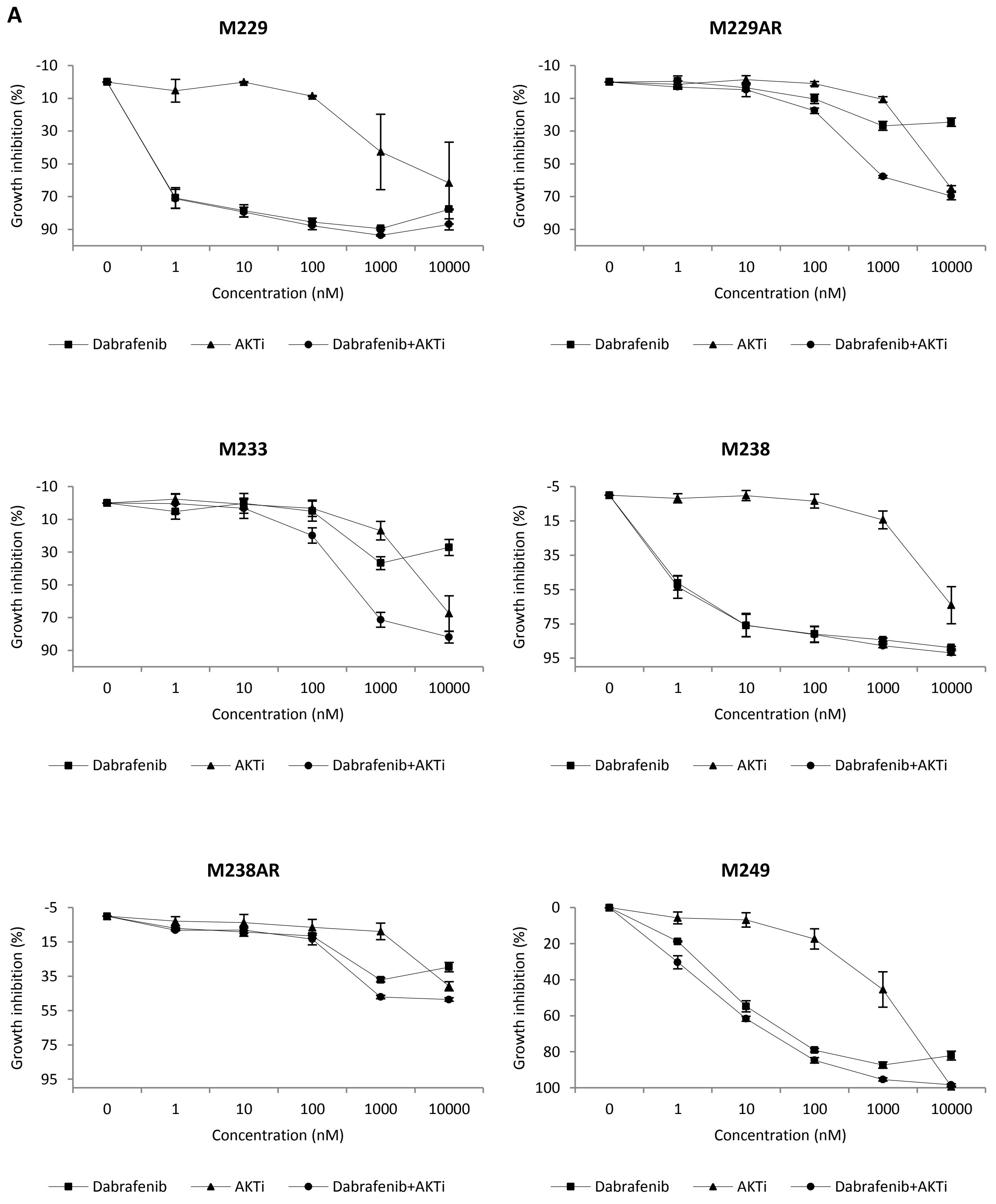
A
M229
M229AR
M233
M238
M238AR
M249
Growth inhibition (%)
Concentration (nM)
0
1
10
100
1000
10000
Dabrafenib
AKTi
Dabrafenib+AKTi

Figure S1
2/4

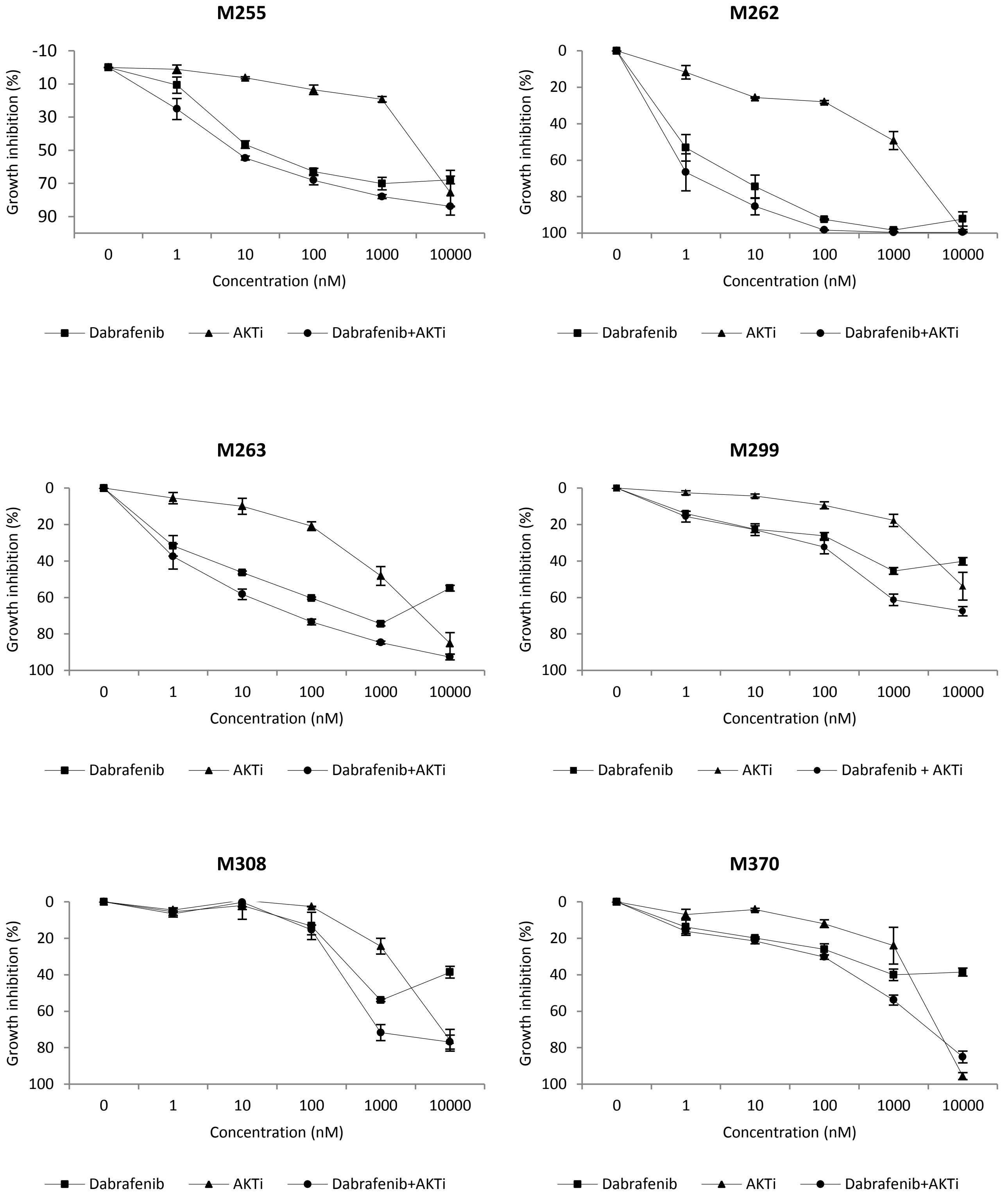
M255
-10
10
30
50
70
90
Growth inhibition (%)
0
1
10
100
1000
10000
Concentration (nM)
Dabrafenib
AKTi
Dabrafenib+AKTi
M262
0
20
40
60
80
100
Growth inhibition (%)
0
1
10
100
1000
10000
Concentration (nM)
Dabrafenib
AKTi
Dabrafenib+AKTi
M263
0
20
40
60
80
100
Growth inhibition (%)
0
1
10
100
1000
10000
Concentration (nM)
Dabrafenib
AKTi
Dabrafenib+AKTi
M299
0
20
40
60
80
100
Growth inhibition (%)
0
1
10
100
1000
10000
Concentration (nM)
Dabrafenib
AKTi
Dabrafenib + AKTi
M308
0
20
40
60
80
100
Growth inhibition (%)
0
1
10
100
1000
10000
Concentration (nM)
Dabrafenib
AKTi
Dabrafenib+AKTi
M370
0
20
40
60
80
100
Growth inhibition (%)
0
1
10
100
1000
10000
Concentration (nM)
Dabrafenib
AKTi
Dabrafenib+AKTi

Figure S1
3/4

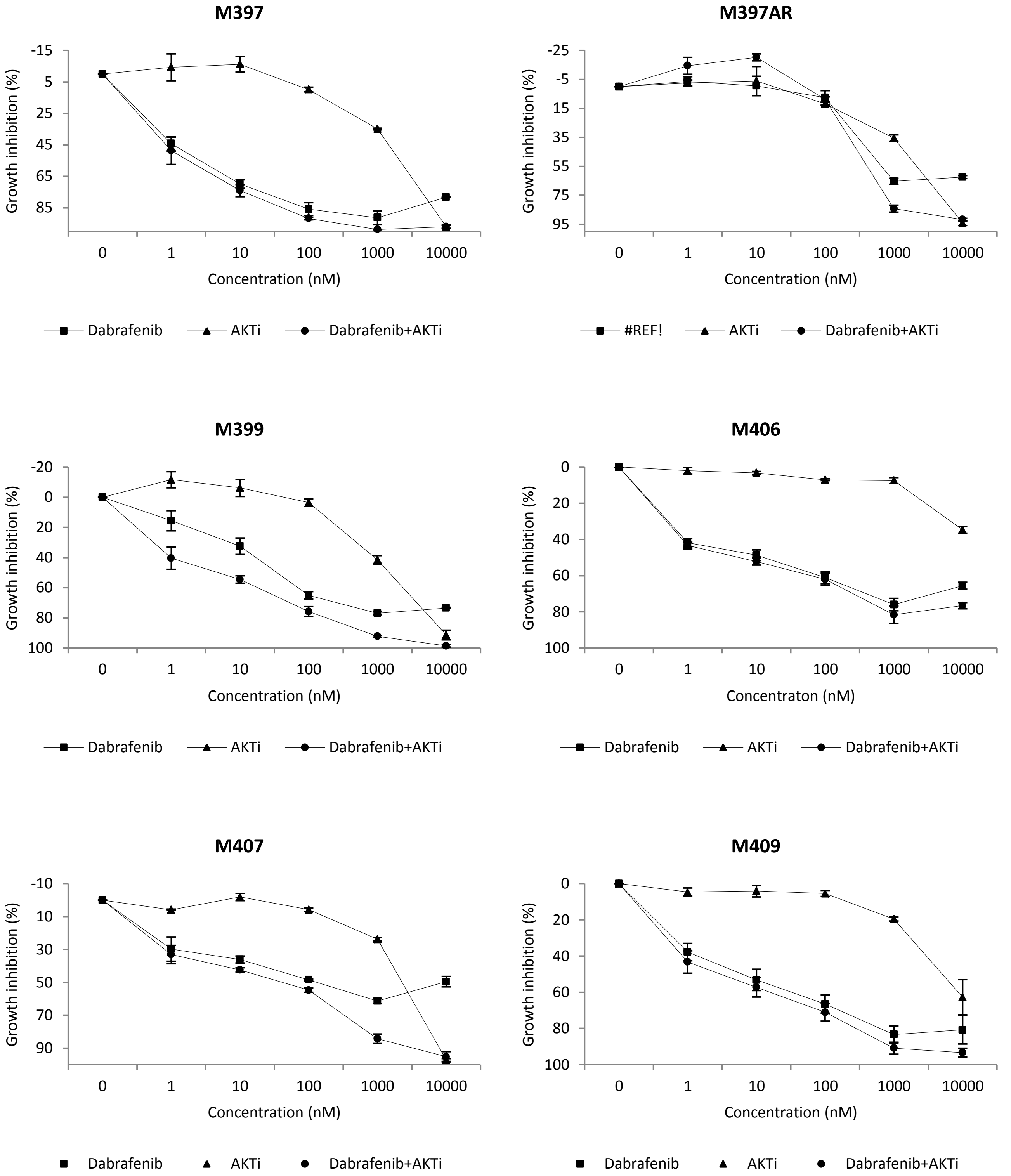
M397
Growth inhibition (%)
-15
5
25
45
65
85
0
1
10
100
1000
10000
Concentration (nM)
Dabrafenib
AKTi
Dabrafenib+AKTi
M397AR
Growth inhibition (%)
-25
-5
15
35
55
75
95
0
1
10
100
1000
10000
Concentration (nM)
#REF!
AKTi
Dabrafenib+AKTi
M399
Growth inhibition (%)
-20
0
20
40
60
80
100
0
1
10
100
1000
10000
Concentration (nM)
Dabrafenib
AKTi
Dabrafenib+AKTi
M406
Growth inhibition (%)
0
20
40
60
80
100
0
1
10
100
1000
10000
Concentraton (nM)
Dabrafenib
AKTi
Dabrafenib+AKTi
M407
Growth inhibition (%)
-10
10
30
50
70
90
0
1
10
100
1000
10000
Concentration (nM)
Dabrafenib
AKTi
Dabrafenib+AKTi
M409
Growth inhibition (%)
0
20
40
60
80
100
0
1
10
100
1000
10000
Concentration (nM)
Dabrafenib
AKTi
Dabrafenib+AKTi

Figure S1
4/4

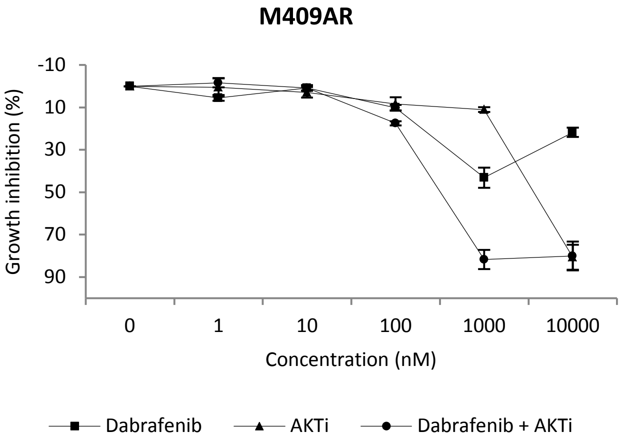

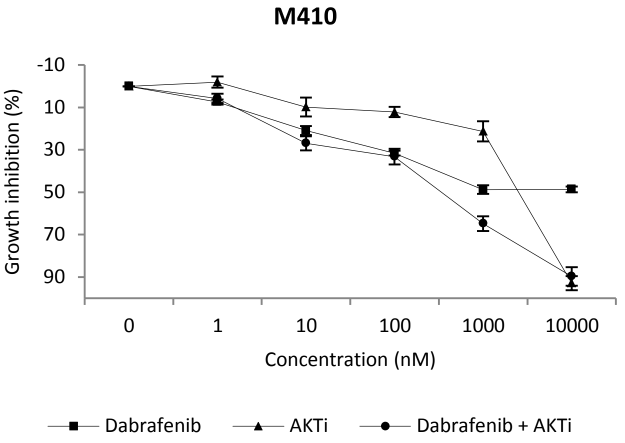

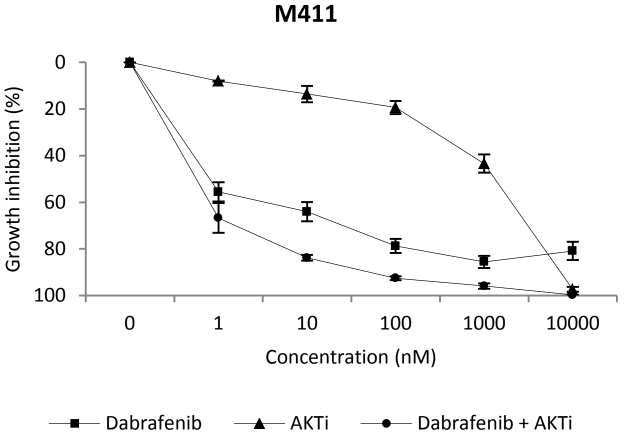

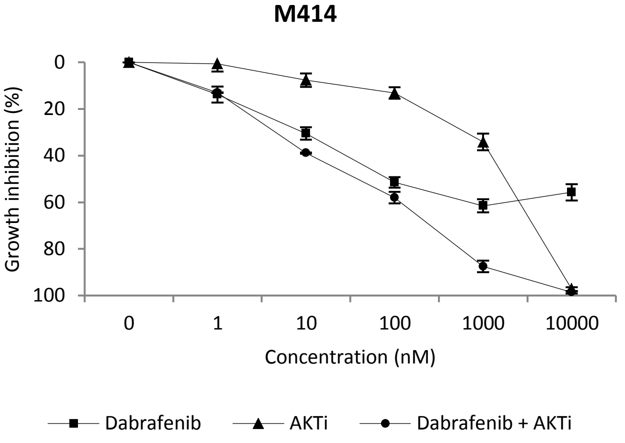

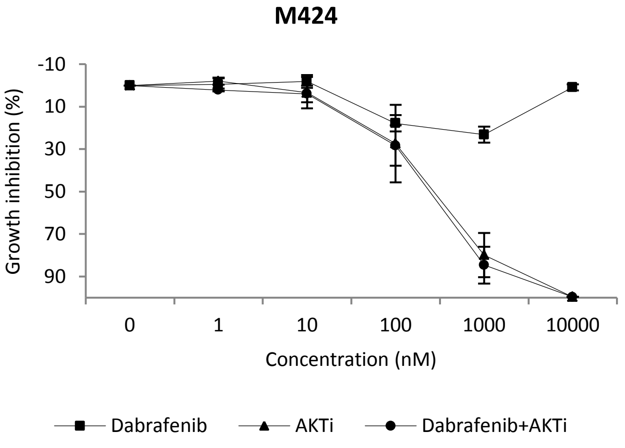

B

**Cell line**	**Dabrafenib**		**AKTi**		**Dabrafenib + AKTi**	
	**m**	**r**	**m**	**r**	**m**	**r**
**M249**	0,36	0,89	0,70	0,84	0,61	0,99
**M399**	0,33	0,92	1,36	0,94	0,70	0,98
**M263**	0,14	0,72	0,68	0,97	0,33	0,99
**M229AR**	0,92	0,89	0,60	0,59	0,73	0,85
**M397AR**	0,80	0,72	1,39	0,93	1,11	0,97
**M238AR**	0,23	0,89	0,55	0,83	0,31	0,91
**M370**	0,18	0,89	0,56	0,84	0,35	0,96
**M233**	0,45	0,93	0,75	0,91	0,88	0,96
**M410**	0,86	0,85	1,23	0,92	0,83	0,95
**M308**	0,47	0,76	0,60	0,77	0,65	0,79
**M409AR**	0,47	0,81	0,86	0,92	1,07	0,94
**M255**	0,35	0,86	1,01	0,92	0,43	0,99
**M414**	0,28	0,88	1,01	0,91	0,69	0,99
**M299**	0,17	0,94	0,41	0,94	0,29	0,96
**M407**	0,36	0,82	1,14	0,83	0,59	0,96
