# Supplementary figures and images for: Effects of AKT inhibitor therapy in response and resistance to BRAF inhibition in melanoma
Source: Mol Cancer. 2014 Apr 16;13:83. doi: 10.1186/1476-4598-13-83 (PMC4021505; doi:10.1186/1476-4598-13-83)

Figure S2

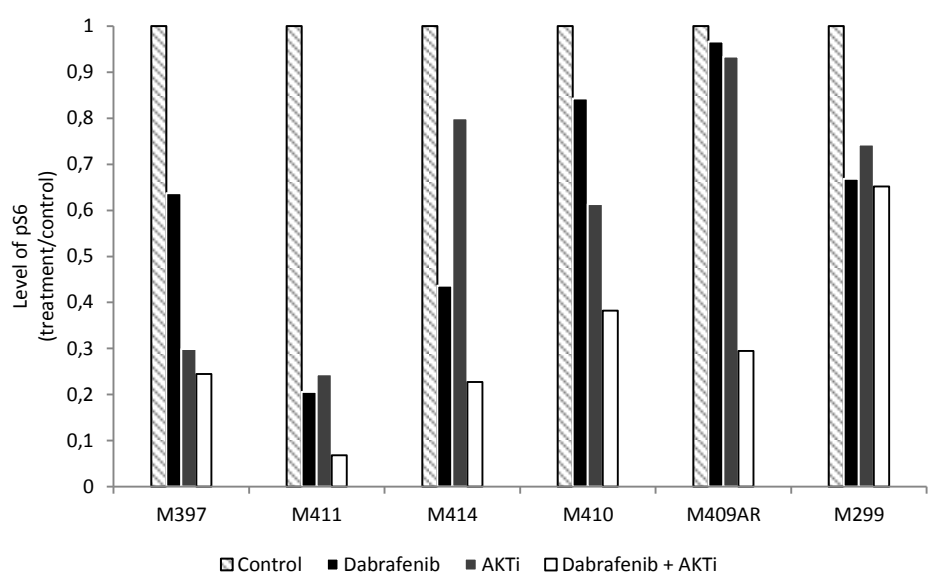

Supplement: Additional file 2: Figure S2 — Quantitative analysis of p-S6 from western blots. Data corresponds to the p-S6 bands in the western blots presented in figure 4. The relative reduction in p-S6 in cell lines M411, M397, M414, M410 and M409AR corresponds with the response to the combined treatment, with the more sensitive cell line M411 showing the highest reduction and the resistant cell line M299 showing the slightest reduction in p-S6. [file 1476-4598-13-83-S2.pdf]

Figure S3

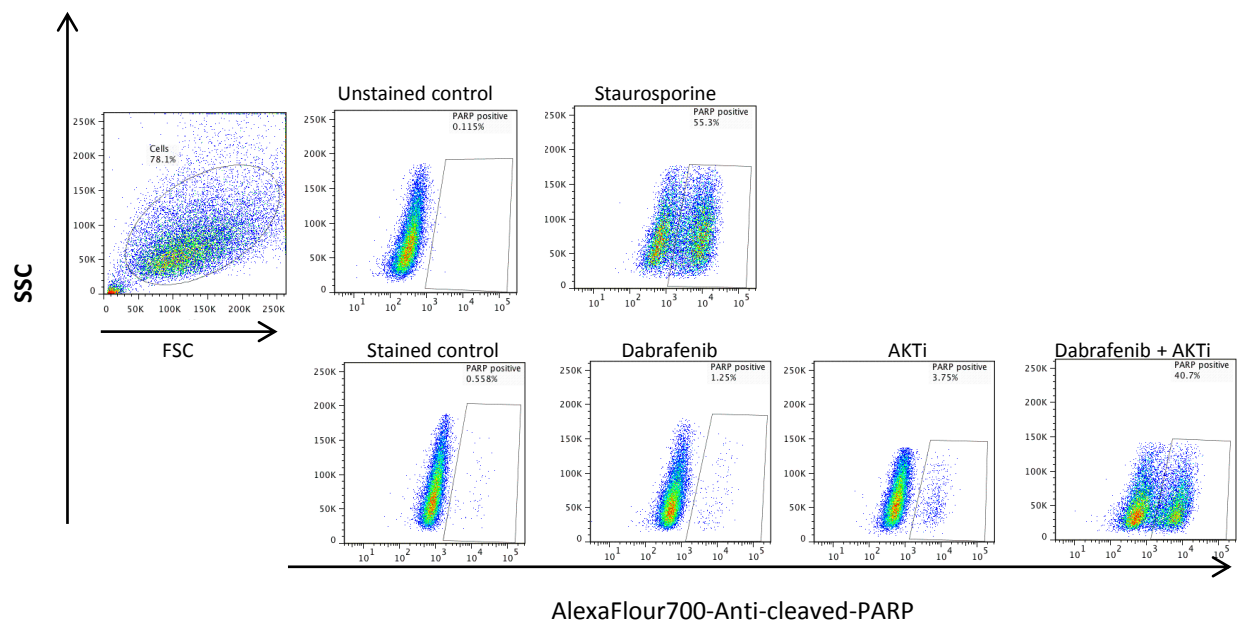

Supplement: Additional file 3: Figure S3 — Anti-cleaved-PARP gating strategy. The fraction of anti-cleaved-PARP positive cells was determined by using an unstained control. Apoptosis induced by staurosporine was used as a positive control. The analysis was done by using FlowJo software (PC ver. 7). [file 1476-4598-13-83-S3.pdf]

Figure S4

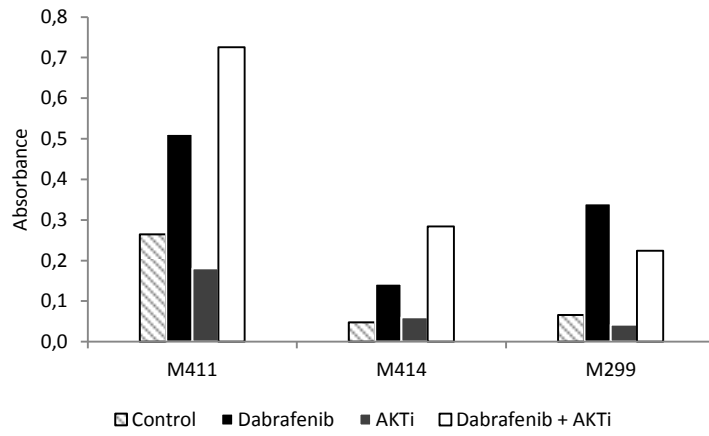

Supplement: Additional file 4: Figure S4 — Determining apoptosis using a cell death detection ELISA. Cells were treated with DMSO, 50 nM dabrafenib, 2.5 μM AKTi or the combination for 48 hours. The extent of apoptosis is reflected by the enrichment of nucleosomes in the cytoplasm, which was quantitated as the relative increase in absorbance (y-axis). The panel included one cell line sensitive to both dabrafenib and AKTi (M411), one cell line sensitive to AKTi but intermediate resistant to dabrafenib (M414) and one cell line demonstrating resistance to both drugs (M299). The bars represent the average absorbance of triplicates. [file 1476-4598-13-83-S4.pdf]

Figure S5

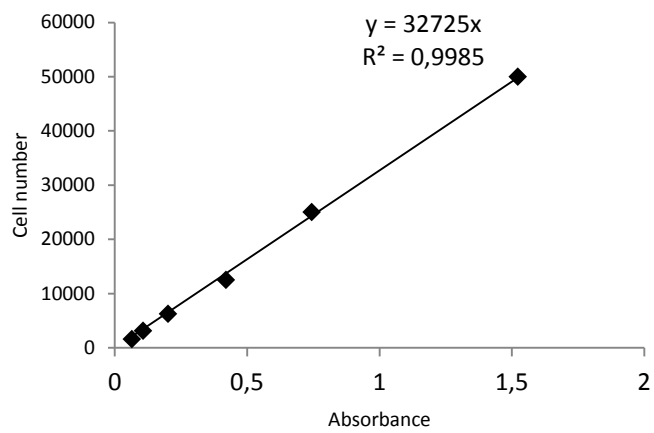

Supplement: Additional file 5: Figure S5 — Example of a standard curve used for the calculations of cell number in long term culture. Cells were plated in 1:2 serial dilutions starting from 50,000 cells. The measured absorbance (using a MTS-based assay) from these wells were plotted against the known cell numbers and by use of the equation for the trend line through (0.0) the unknown cell numbers could be determined. [file 1476-4598-13-83-S5.pdf]
